# Emerging Trends and Promises in Orofacial Cancers

**DOI:** 10.3389/fphys.2019.00679

**Published:** 2019-05-29

**Authors:** Thimios A. Mitsiadis

**Affiliations:** Orofacial Development and Regeneration, Institute of Oral Biology, Centre for Dental Medicine, University of Zurich, Zurich, Switzerland

**Keywords:** head and neck squamous cell carcinomas, oral cancers, Notch signaling, cancer stem cells, cancer drugs, organs-on-chips, organoids

Orofacial cancers result in facial deformities and impairment of vital functions and are often lethal. These aggressive solid tumors exhibit great heterogeneity between them and show distinct and exclusive molecular alterations that deregulate the function of important signaling pathways. The Notch signaling pathway is involved in the initiation and development of orofacial cancers. Increasing evidence suggests that Notch molecules may have a dual function in cancer, acting either as oncogenes or tumor suppressor genes. Crosstalk between Notch and other signaling pathways provides a critical multidirectional control in these cancers. Protein phosphorylation is activated in cancers and therefore novel drugs inhibiting phosphorylation events (kinase inhibitors) are increasingly used in the treatment of cancers. Another pharmacological strategy is the selective targeting of Notch signaling in order to eliminate the cancer stem cells using monoclonal antibodies against specific regions of the Notch molecules. Organoids, “organ-on-a-chip” devices and single-cell genomic analyses could be used for further investigations and preclinical studies in orofacial cancers. Organoids can be used to study complex interactions between the various cells lines in orofacial cancers, as well as for the preclinical screening of novel drugs. Microfluidic culture systems, also called “organs-on-chips,” can be used to model cancer cell behavior within orofacial tissues and their environment. These chips also enable to vary drug delivery and composition in a controlled manner in order to study cancer tissue responses to various pharmaceutical anticancer products. Single-cell RNA-seq analyses allow exploring the genetic and functional heterogeneity of orofacial cancers at a cellular resolution, thus revealing new insights into tumor composition and drug resistance. These important technological developments and the innovative therapeutic strategies demonstrate significant promise and generate enthusiasm and optimism within the oncology community.

## Genetic Alterations and Notch Signaling in Orofacial Cancers

Head and neck squamous cell carcinomas (HNSCCs) are the sixth most common cancers diagnosed worldwide and can result in facial deformities and impairment of vital functions such as swallowing, speech, and taste (Porcheri et al., 2019). These solid tumors are aggressive and often lethal (the eighth most common cause of cancer death) with a 5-years survival rate of 50% of the cases (Nyman et al., 2018). The use of tobacco and excessive consumption of alcohol are major risk factors for HNSCCs. Human papillomavirus (HPV) is an additional risk factor for the development of HPV-associated HNSCCs. HPV-associated tumors exhibit distinct biological features and patients have an overall improved survival rate (Nyman et al., 2018). HNSCCs principally arise from a pre-neoplastic dysplasia that undergoes a series of genetic and epigenetic modifications to progress toward malignancy. Although morphologically similar, HNSCCs exhibit distinct and exclusive molecular alterations that deregulate the function of important signaling pathways. HNSCCs exhibit many chromosomal abnormalities such as amplifications of region 11q13 that contains the *cyclin D1* gene and region 7p11 that encodes *epidermal growth factor receptor* (*EGFR*) (Agrawal et al., 2011).

An involvement of *NOTCH1* in human cancers was first demonstrated through the discovery of translocations in T cell leukemias (Ellisen et al., 1991). Subsequently, more subtle mutations of *NOTCH1* were identified in a variety of hematopoietic and solid tumors (Grabher et al., 2006). The Notch signaling pathway is evolutionary conserved and required for cell fate decisions during embryogenesis and tissue regeneration (Blanpain et al., 2006; Mitsiadis and Graf, 2009; Artavanis-Tsakonas and Muskavitch, 2010). Notch signaling is activated when the membrane-bound Notch receptors (Notch-1, -2, -3, and -4) interact with their specific membrane-bound ligands (Jagged-1, -2, Delta-like-1, -3, -4) on adjacent cells. In the oral cavity, members of the Notch pathway are mainly confined to the oral mucosa (Mitsiadis et al., 1995, 1997). Loss of NOTCH1 in this tissue leads to oral epithelial dysplasias and promotes tumor initiation while impairing barrier integrity and generating a wound-like environment in the underlying stroma (mesenchyme) (Sakamoto, 2016; Nyman et al., 2018). Numerous other findings have also shown that reduced activity of other components of the Notch pathway is associated with the development of various cancers. Taken together, these observations suggest that Notch signaling possesses a tumor suppressor function rather than having an oncogene function. In contrast, the large number of mutations in hematopoietic tumors strongly implicates Notch molecules as tumor drivers. However, the NOTCH1 mutations that have been observed in HNSCCs were localized in the N-terminal epidermal growth factor (EGF)-like ligand-binding domain and therefore are different from those mutations identified in hematopoietic tumors. The location and nature of these alterations provide solid genetic evidence that *NOTCH1* acts mainly as a tumor suppressor gene in HNSCCs (Agrawal et al., 2011; Stransky et al., 2014; Lawrence et al., 2015). Indeed, experiments in genetically modified mice with *Notch1* deletion in epithelium have demonstrated that these animals develop epithelial tumors (Nicolas et al., 2003). Therefore, NOTCH1 may have a dual function in cancer, acting either as an oncogene in several leukemia cases or as a tumor suppressor gene in HNSCCs (Yap et al., 2015; Porcheri et al., 2019).

Bioinformatics analyses on HNSCCs have identified *Notch1* as one of the genes (Lawrence et al., 2015), together with several coding for receptor tyrosine kinases, that are frequently affected by genomic alterations. Crosstalk of Notch signaling with other molecular pathways is important in regulating proliferative, apoptotic, and invasive processes (Porcheri et al., 2019). Notch interacts with p53, a tumor suppressor protein that can be activated by diverse stressful signals to ultimately modulate cellular responses such as transient cell cycle arrest, senescence and apoptosis (Porcheri et al., 2019). Experiments in animal models have shown reduced p53 levels upon Notch1 activation (Porcheri et al., 2019). Crosstalk between Notch and Wnt signaling occurs in various cancers where β-catenin/T-cell factor/lymphoid enhancer factor (TCF) activates Notch signaling that leads to the expression of Myc proto-oncogene (Myc) (Porcheri et al., 2019). Activation of the Wnt pathway can occur in absence of β-catenin in HNSCCs. Sonic Hedgehog (SHH) signaling is also upregulated in HNSCCs and mutations in this pathway have been found in basal cell carcinomas. SHH increases the transcription of genes involved in the Notch pathway, while Notch regulates the intracellular localization of SHH (Porcheri et al., 2019). Collectively, these findings suggest a critical multidirectional control of the various signaling pathways within tumors, thus providing new insights that might accelerate the progress in HNSCCs prevention and therapy.

## Innovative Therapeutic Strategies for Orofacial Cancers

The different genetic landscapes, associated with tobacco exposure, alcohol and HPV, are consistent with clinical and epidemiologic data. These factors are important in prognosis and response to therapeutic interventions (Porcheri et al., 2019). Signaling networks that employ phosphorylation to regulate cell activities are involved in cancer, where abnormal activation of protein phosphorylation is frequently either a driver or direct consequence of this disease (Gross et al., 2015). Kinase signaling pathways are involved in many activities of tumor tissues such as cell proliferation and motility, as well as antitumor immune responses. Therefore, novel therapeutic approaches are focused in developing drugs to inhibit protein phosphorylation. Small molecule kinase inhibitors with suitable pharmaceutical properties are increasingly used in the treatment of cancers (Gross et al., 2015). New oncology drugs that also target kinases have been approved, and ongoing clinical trials are in various stages of evaluation. Another therapeutic opportunity may exist in modulating tumor immunity, which represents a key mechanism in HNSCCs development (Michot et al., 2016; Elicin et al., 2019). This important therapeutic strategy has demonstrated significant promise within the cancer field. While kinases play central roles in immune responses and could therefore serve as relevant therapeutic tools, this field is still in its infancy.

Cancer stem cells, which exhibit self-renewal and tumor-initiating properties, are involved in chemotherapeutic drug resistance. Deregulation of key epigenetic cues can shift the balance toward oncogenic transformation, leading to development and metastasis of HNSCCs. Understanding of this epigenetic balance is critical for valid drug design and accurate therapy. Epigenetic drugs (epidrugs) could be used in combination with chemotherapy or immunotherapy in order to reprogram cancer stem cells and sensitize to chemotherapy (Albiges et al., 2015; Bais, 2019). Dysregulation of EGF activates the immune system in HNSCCs, thus suggesting that EGF mediators, epidrugs and chemotherapy could be used in synergy for regulating immune responses, thus offering suitable and more appropriate therapeutic outcomes for HNSCCs (Bais, 2019).

Notch is a key player in regulating cancer stem cell maintenance and mobility during metastasis (Artavanis-Tsakonas and Muskavitch, 2010; Porcheri et al., 2019). Therefore, one of the main pharmacological strategies is the selective targeting of Notch signaling in order to eliminate the cancer stem cells. Monoclonal antibodies either targeting specific regions of the Notch ligand-receptor binding domain or blocking the nuclear translocation of Notch (such as the molecule GSI DAPT) have been used in combined cancer treatments. This treatment, as well as the combinatory use of Notch inhibitors and chemotherapeutic drugs, resulted in a significant reduction of cancer stem cells (Porcheri et al., 2019). However, the use of these Notch-tailored molecules may cause severe side effects and therefore requires periodic administration with lag phases for patient recovery.

## Potential Use of Novel Technological Developments in Orofacial Cancer Research

Although these recent findings have contributed to a certain molecular understanding of HNSCCs regulation, further investigations on the differences between HNSCCs and other cancer types and subtypes would surely increase the chances to treat better this disease. Genetically modified and humanized mouse models have been used to test epigenetic regulators and other candidates in HNSCCs (Porcheri et al., 2019). Recently developed tools such as organoids (Rossi et al., 2018), “organ-on-a-chip” devices (Sontheimer-Phelps et al., 2019), and single-cell genomic analyses (Puram et al., 2017) could be used for further research and preclinical studies concerning HNSCCs. Organoids are three-dimensional (3D) structures of different organ-specific cell types derived from the culture of primary stem cells that share many structural and molecular features of the organ of interest (Baker, 2018; Rossi et al., 2018). Organoids can emulate normal or pathological organ structures and thus can be used to study complex interactions between the various cells types in physiological and cancer conditions. Organoids generated from patient samples can be genetically engineered, thereby generating a unique system to study HNSCCs initiation and progression. Organoids can be also used for the preclinical screening of novel drugs against these tumors. Recent developments in microfluidic culture systems have led to the generation of human “organs-on-chips” that can be used to model cancer cell behavior within orofacial tissues and their microenvironment (Sontheimer-Phelps et al., 2019). These chips enable to vary cellular, molecular and pharmaceutical cues in a controlled manner while analyzing how these parameters contribute to HNSCCs initiation, progression and responses to various drug therapies. Advances in single-cell genomics provide an exceptional tool to explore genetic and functional HNSCCs heterogeneity at a cellular resolution (Puram et al., 2017). These single-cell RNA-seq studies have revealed new insights into tumor composition, cancer stem cells and drug resistance. Epithelial-to-mesenchymal transition (EMT) markers have been detected and associated with cancer metastatic events.

## Conclusion

All these exciting recent developments generate enthusiasm and optimism within the medical community that selective targeted HNSCCs therapies will continue to improve and significantly extend patients' lives.

## Author Contributions

The author confirms being the sole contributor of this work and has approved it for publication.

### Conflict of Interest Statement

The author declares that the research was conducted in the absence of any commercial or financial relationships that could be construed as a potential conflict of interest.
